# Lipid Composition-Driven Colloidal Transformation from Hexosomes to Nanoparticles with Highly Disordered Internal Nanostructures in Monolinolein/Dilinolein Nanodispersions

**DOI:** 10.3390/molecules31173031

**Published:** 2026-08-28

**Authors:** Gokce Dicle Kalaycioglu

**Affiliations:** 1 Department of Chemical Engineering, Hacettepe University, Beytepe, 06800 Ankara, Turkey; gokcedicle.demir@hacettepe.edu.tr; 2 Department of Pharmacy, Faculty of Health and Medical Sciences, University of Copenhagen, Universitetsparken 2, DK-2100 Copenhagen, Denmark

**Keywords:** cryogenic transmission electron microscopy, dilinolein, hexosomes, inverse hexagonal phase, monolinolein, Synchrotron small-angle X-ray scattering

## Abstract

Nonlamellar liquid crystalline nanodispersions produced from single monoacylglycerols or from their combinations with fatty acids or other amphiphiles have attracted interest owing to their structural versatility and tunability. In this study, we investigated the effect of dilinolein (DLO) incorporation on the structural features of Pluronic F127-stabilized monolinolein (MLO) nanodispersions using small-angle X-ray scattering (SAXS), cryogenic transmission electron microscopy (cryo-TEM), and dynamic light scattering (DLS). We report a lipid composition-dependent direct colloidal transformation from hexosomes, defined as nanoparticles with an ordered internal inverse hexagonal (H_2_) phase, toward nanoparticles with highly disordered internal nanostructures upon the partial replacement of MLO by DLO. Small-angle X-ray scattering (SAXS) measurements showed that, at relatively low DLO content, the MLO-rich MLO:DLO 90:10 (*w*/*w*) nanodispersion retained an ordered internal H_2_ phase, with three well-defined characteristic Bragg peaks, whereas the SAXS patterns recorded for nanodispersions containing ≥20 wt% DLO displayed loss of the characteristic H_2_ Bragg reflections and broad correlation maxima, indicating loss of long-range internal periodicity and the emergence of highly disordered internal nanostructures. Similarly, the control nanodispersion prepared from DLO alone displayed a broad, low-intensity correlation maximum rather than distinct Bragg peaks, consistent with a highly disordered internal nanostructure. For the nanodispersions displaying broad SAXS correlation maxima, the SAXS-derived characteristic distance decreased monotonically from 4.53 to 2.87 nm as the DLO fraction increased. Additional cryo-TEM observations of two nanodispersions containing relatively high DLO contents revealed predominantly spherical nanoparticles characterized by an intense lipid core and discernible internal nanoscale features. However, these internal features were not sufficiently resolved to allow full characterization and unambiguous assignment of the internal phase, consistent with a highly disordered inverse nanostructure that may include an L_2_-like inverse-micellar organization alongside other possible arrangements such as nanoemulsion droplets. Taken together, the SAXS and cryo-TEM observations indicate a composition-driven colloidal transformation from hexosomes with a well-defined internal H_2_ phase toward nanoparticles with a highly disordered internal phase as the DLO content increases. The experimental findings suggest that DLO modifies lipid packing at the MLO–water interface and promotes more negative spontaneous curvature, favoring the observed structural transformation. The ability to tune the internal nanostructure by lipid composition while maintaining nanoscale particle size and low dispersity makes these nanodispersions attractive platforms for drug nanocarrier development.

## 1. Introduction

Certain amphiphilic lipids can self-assemble in water into lyotropic liquid-crystalline (LLC) phases, which have attracted long-standing interest in soft matter, colloid science, and drug delivery, owing to their structural versatility and ability to encapsulate compounds with different physicochemical properties [1,2,3,4,5,6,7,8,9,10,11]. Depending on their molecular geometries and experimental conditions, including hydration state, temperature, and pressure, these amphiphiles can self-organize into lamellar (L_α_) or inverse nonlamellar phases, including bicontinuous cubic (Q_2_) and inverse hexagonal (H_2_) phases, or into inverse micellar solutions (L_2_ phases) [12,13,14,15,16]. These phases can be dispersed in excess water in the presence of a suitable stabilizer to produce colloidal internally self-assembled nano-objects. Among these, cubosomes, hexosomes, and micellar cubosomes are the most extensively investigated nanodispersions [3,9,11,17]. Owing to their nanoscale dimensions and structurally versatile internal architectures with large interfacial areas, they are attractive platforms for drug and functional food delivery applications, where they may enhance the loading of bioactive compounds and modulate their release properties [9,16,18,19]. In addition to temperature, lipid type and composition play important roles in modulating the internal nanoarchitecture of cubosomes, hexosomes, micellar cubosomes, and related internally self-assembled nanodispersions by altering lipid packing, interfacial hydration, and the preferred spontaneous curvature of the lipid–water interface [20,21,22].

Monoacylglycerols (MAGs) remain among the most versatile amphiphiles of biological relevance for generating inverse nanostructures [16,21]. Monolinolein (MLO), a MAG containing a linoleoyl chain (C18:2), is structurally related to monoolein (MO), the most widely investigated monounsaturated monoacylglycerol with a strong propensity to form nonlamellar liquid-crystalline phases in excess water. Compared with fully hydrated MO, MLO undergoes similar phase transitions at lower temperatures, making it attractive for forming nonlamellar liquid-crystalline phases and their fragmentation into internally structured nanodispersions under milder conditions [23,24,25]. Accordingly, MLO-based and related MAG nanodispersions have been investigated in biophysical studies, while their structurally tunable internal architectures make them attractive for drug delivery, functional food delivery, and biomedical applications [26,27]. Controlled changes in lipid composition may therefore provide a useful strategy for modulating the confined internal structural architectures of MLO-based nanoparticles.

Dilinolein (DLO), a diacylglycerol (DAG) containing two linoleoyl chains (C18:2), differs from MLO in its molecular packing and stronger tendency to promote negative spontaneous curvature at the lipid–water interface. Compared with MAGs, DAGs possess smaller effective headgroup areas and larger hydrophobic volumes, which favor inverse self-assembled structures in excess water [28,29,30,31]. DAGs are further characterized by a large critical packing parameter and pronounced negative spontaneous curvature (L_2_) topology, a necessary but insufficient condition for inverse-micellar organization (as opposed to inverse-hexagonal or bicontinuous-cubic) [20,32,33]. This behavior makes DLO a relevant lipid component for examining how DAG incorporation affects the confined internal phase behavior of MLO-based nanodispersions. Although studies specifically addressing the effect of partial replacement of MAGs with DAGs in lipid nanodispersions remain limited, previous investigations have shown that the internal nanostructure of MAG-based systems can be effectively modulated by incorporating DAGs, fatty acids, or other lipid components [10,15,34,35]. Depending on formulation composition, these systems can undergo structural transitions between bicontinuous cubic, inverse hexagonal, inverse micellar cubic (*Fd3m*), and L_2_ phases [10,15,35]. However, systematic investigations of stabilized aqueous nanodispersions based on fully polyunsaturated MAG/DAG combinations remain limited. Combining MLO and DLO therefore provides a useful platform for probing how lipid composition influences colloidal organization. Under controlled formulation conditions, gradual substitution of MLO with DLO is expected to progressively alter lipid packing and interfacial curvature, thereby inducing transitions between ordered (H_2_) and disordered (L_2_) inverse structures. Accordingly, the objective of this study was to investigate how systematic variation in the MLO:DLO ratio influences the internal nanostructure and colloidal behavior of these dispersions.

In this study, we report how DLO incorporation affects the internal phase behavior, morphology, and size of nanodispersions produced from binary MLO/DLO mixtures over a series of lipid compositions from MLO:DLO 90:10 to 30:70 (*w*/*w*), together with a control nanodispersion prepared from DLO alone, at fixed total lipid and Pluronic F127 concentrations. Synchrotron small-angle X-ray scattering (SAXS) and cryogenic transmission electron microscopy (cryo-TEM) were used as complementary characterization techniques. Together, the results revealed a composition-dependent colloidal transformation from hexosomes with a well-defined internal H_2_ phase toward nanoparticles with highly disordered internal nanostructures. These findings provide insight into how DLO incorporation modulates the confined internal organization of MLO-based nanodispersions. Such composition-guided control of internal nanostructure may support the rational design of structurally tunable lipid nanocarriers for drug delivery.

## 2. Results and Discussion

### 2.1. Structural Characterization of Pluronic F127-Stabilized MLO/DLO Nano-Self-Assemblies

**Figure 1:**
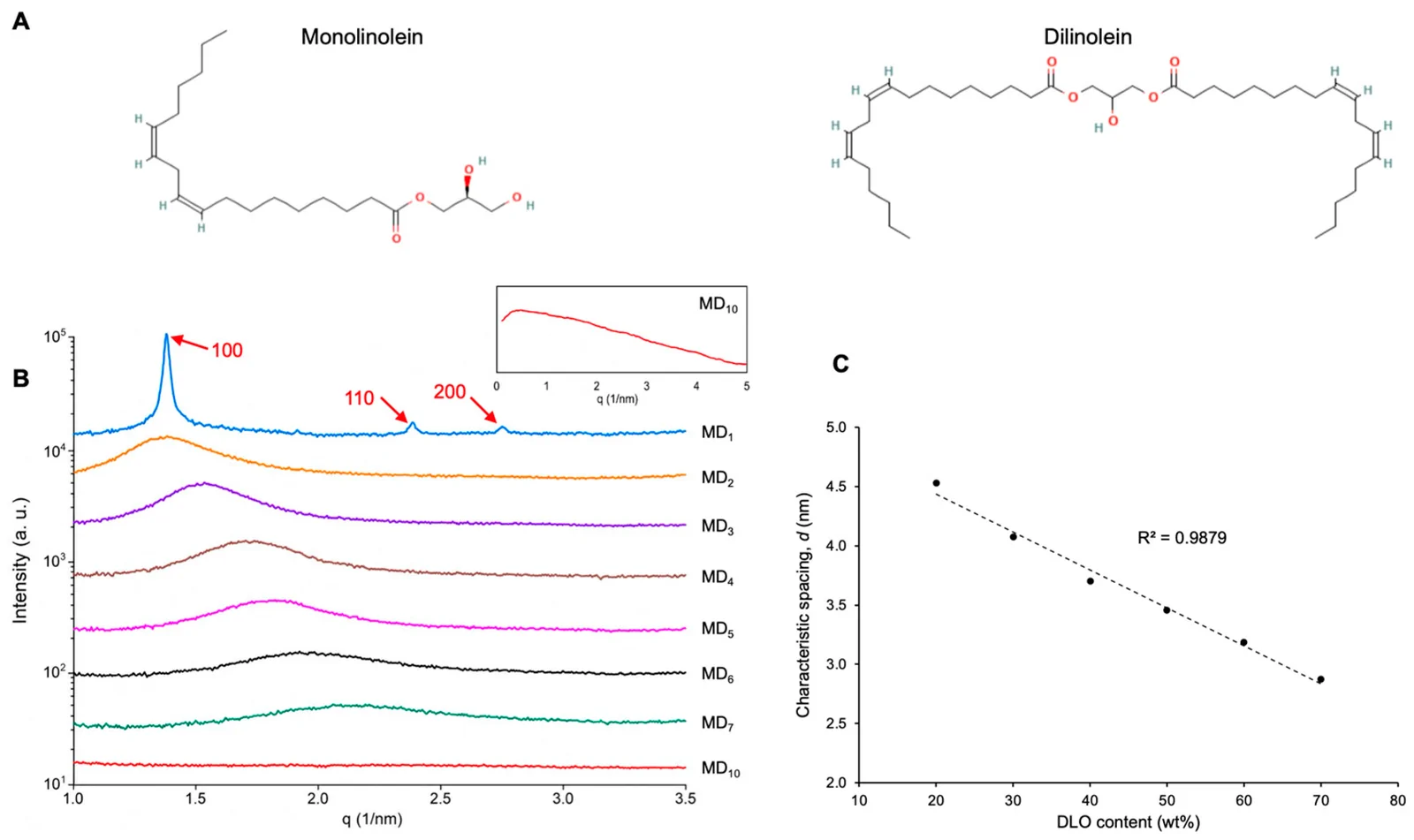
(**A**) Molecular structures of monolinolein (MLO) and dilinolein (DLO). (**B**) Synchrotron SAXS profiles of Pluronic F127-stabilized MLO/DLO nanodispersions prepared at different lipid compositions. The nanodispersions were prepared at the following MLO:DLO weight ratios (*w*/*w*): MD_1_, 90:10; MD_2_, 80:20; MD_3_, 70:30; MD_4_, 60:40; MD_5_, 50:50; MD_6_, 40:60; MD_7_, 30:70; and MD_10_, 0:100. (**C**) Composition-dependent variation in the characteristic distance, *d*, of the internal L_2_ phases in MLO/DLO nanodispersions as a function of DLO content. The *d* value was calculated from the position of the SAXS correlation maximum using *d =* 2*π*/*q*_max_. The inset in panel B shows the complete SAXS profile of the MD_10_ nanodispersion over the measured *q*-range.

Synchrotron SAXS was used to examine how progressive replacement of MLO by DLO affects the confined internal self-assembled architectures of the nanodispersions. The molecular structures of MLO with DLO are presented in Figure 1A. At the highest MLO content, the scattering profile of the MLO:DLO 90:10 (*w/w*) nanodispersion (sample MD_1_) displayed three characteristic Bragg peaks [(100), (110), and (200), marked with red arrows in Figure 1B] with relative peak positions following the characteristic 1:√3:√4 ratio of an inverse hexagonal (H_2_) phase indicating the formation of hexosomes. The corresponding lattice parameter of this internal H_2_ phase, calculated from the position of the first-order Bragg peak, was approximately 5.27 nm. The calculated lattice parameter is consistent with values previously reported for Pluronic F127-stabilized MLO-based hexosomes [35,36]. The sharpness and relative intensity of the Bragg reflections suggest that MLO-rich binary lipid mixture provides a sufficiently hydrated and flexible interfacial environment to stabilize this inverse phase [23].

Increasing the DLO fraction induced a composition-dependent colloidal transformation from hexosomes, characterized by an ordered internal H_2_ phase, toward nanoparticles with highly disordered internal nanostructures (Figure 1B). In the nanodispersions containing 20–70 wt% DLO (samples MD_2_–MD_7_), the Bragg peaks assigned to the H_2_ phase disappeared and were replaced by a single broad correlation peak, indicating loss of long-range H_2_ order and the presence of short-range structural correlations within a highly disordered internal nanostructure. Although such broad correlation maxima may be compatible with an L_2_-like inverse-micellar organization, they are not, by themselves, sufficient for an unequivocal assignment of the internal phase. The absence of Bragg reflections is not by itself diagnostic of a specific structural state: it is equally consistent with an inverse-micellar (L_2_) phase, a normal micellar or nanoemulsion-droplet morphology, or other unstructured inverse aggregates. Throughout this manuscript, ‘L_2_’ is used as a descriptive term for a highly disordered internal inverse nanostructure lacking long-range periodicity, representing the interpretation most consistent with the combined SAXS and cryo-TEM observations (see Section 2), rather than an unambiguously established phase identity. The SAXS profile of the control nanodispersion based on DLO alone (sample MD_10_, MLO:DLO 0:100, *w*/*w*) displayed a broad, low-intensity correlation maximum rather than a distinct Bragg reflection, evident only when the full *q*-range spectrum is examined with appropriate intensity scaling (see inset, Figure 1B), consistent with a highly disordered internal nanostructure lacking long-range periodicity. The position of the correlation maximum systematically shifted toward higher *q*-values as the fraction of DLO increased, leading to a monotonic decrease in the SAXS-derived characteristic distance, calculated as *d =* 2*π*/*q*_max_, from 4.53 nm for the MLO:DLO 80:20 (*w*/*w*) nanodispersion to 2.87 nm for the MLO:DLO 30:70 (*w*/*w*) nanodispersion. As summarized in Figure 1C, this decrease follows an approximately linear trend (R^2^ = 0.9879) with increasing DLO content, indicating a progressive reduction in the characteristic length scale of the internal L_2_ phase.

The monotonic decrease in the SAXS-derived characteristic distance associated with the highly disordered internal nanostructures with increasing DLO content is most likely associated with reduced water accommodation within the internal nanostructure. Similar water expulsion has been reported during temperature-induced colloidal transformations of Pluronic F127-stabilized MLO nanodispersions from cubosomes and hexosomes toward emulsified L_2_ phase nanodispersions [26,36].

The pronounced impact of DLO on the structural features of MLO nanodispersions can be understood in terms of its molecular packing characteristics. DLO is a diacylglycerol containing two linoleoyl chains and a comparatively small effective polar headgroup area (Figure 1A). Relative to MLO, the additional linoleoyl chain in DLO increases the hydrophobic volume relative to the polar headgroup area, thereby shifting the self-assembled nanostructure in excess water toward stronger inverse spontaneous curvature. DLO is expected to possess a relatively large packing parameter typical of diacylglycerol-like lipids, which are known to promote negative interfacial curvature and nonlamellar organizations [28]. Consistent with the reported phase behavior of other diglycerides, this molecular packing favors negative interfacial curvature consistent with a disordered inverse (L_2_-type) organization under fully hydrated conditions [34,37,38].

The critical packing parameter (CPP), also referred to as the molecular shape factor, is commonly expressed as *CPP* = *v*/(*a.l*), where *v* is the hydrophobic chain volume, *a* is the effective headgroup area, and *l* is the hydrophobic chain length of the amphiphile [20]. CPP provides a useful framework for describing lipid self-assembly behavior and spontaneous curvature tendencies. Amphiphilic lipids with CPP values significantly greater than unity generally favor inverse self-assembled nanostructures; however, the specific phase depends on the magnitude of the negative spontaneous curvature of the lipid–water interface. Moderate negative curvature can support inverse bicontinuous cubic and discontinuous H_2_ phases, whereas stronger negative spontaneous curvature, together typically with reduced water accommodation, favors discontinuous inverse-micellar nanostructures, including inverse micellar cubic *Fd3m* phases and inverse micellar L_2_ phases [22,39]. Accordingly, alterations in lipid composition can modulate the effective CPP of mixed amphiphilic systems and thereby influence their preferred mesophase structure. Increasing the DLO fraction in MLO-based nanodispersions is therefore expected to increase the effective CPP and promote more negative spontaneous curvature at the lipid–water interface, favoring loss of the ordered H_2_ phase and a transition toward highly disordered inverse nanostructures. Such structural evolution may involve an L_2_-like inverse-micellar organization at sufficiently high DLO contents.

The disordered inverse nanostructures proposed here for the DLO-rich compositions should not be understood as equilibrium bulk L_2_ phases, which for DAG-containing systems are typically reported at substantially lower water content than the > 90% water used throughout this study. Rather, these nanostructures are considered to be kinetically stabilized and confined within individual nanoparticles: during probe-sonication dispersion (Section 3.2), the lipid mixture is rapidly fragmented and stabilized against macroscopic phase separation by the Pluronic F127 interfacial layer, a non-equilibrium process that can allow an inverse-curvature internal organization to persist within the dispersed nanoparticles even at water contents where the corresponding bulk phase would not be thermodynamically stable.

The alternative possibility of a normal (positive-curvature) micelle can be excluded on molecular-geometry grounds. DLO possesses two linoleoyl chains and a minimal effective headgroup area, corresponding to a critical packing parameter substantially greater than unity [20], a molecular shape that is geometrically incompatible with the positive interfacial curvature required for normal micelle formation, which instead requires a comparatively large headgroup relative to hydrophobic volume, typical of single-chain amphiphiles. This is consistent with the phase behavior reported for closely related DAG-containing systems, where increasing DAG content drives the phase sequence progressively toward more negative curvature (lamellar → bicontinuous cubic → inverse hexagonal → inverse micellar) rather than toward a normal micellar phase [34]. On this basis, a normal micelle is considered an implausible structural outcome for the DLO-rich compositions studied here.

The observed colloidal transformation from hexosomes toward nanoparticles with highly disordered internal nanostructures is consistent with earlier studies on Pluronic F127-stabilized MLO-based nanodispersions [26,36]. Temperature-induced transitions from cubosomes to hexosomes and finally to emulsified L_2_-phase nanodispersions have been reported for MLO nanodispersions, while solubilization of tetradecane in Pluronic F127-stabilized MLO nanodispersions was also shown to promote colloidal transformations from cubosomes to hexosomes and then to emulsified L_2_-phase nanodispersions. Although both tetradecane and DLO can shift the internal self-assembled nanostructures of MLO-based nanodispersions toward internal L_2_ phases, they are expected to act through different modes of incorporation. Tetradecane, as a purely hydrophobic additive, tends to localize within the hydrophobic domains of the internal self-assembled nanostructure, whereas DLO, as an amphiphilic compound, is expected to intercalate into the MLO–water interfacial region. Thus, DLO changes the amphiphile composition of the interfacial film itself, increasing the effective CPP and promoting more negative spontaneous curvature at the lipid–water interface. This provides a composition-induced pathway toward internal L_2_ phases that is analogous to, but distinct from, hydrophobic-additive-induced colloidal transformations in Pluronic F127-stabilized MLO/tetradecane nanodispersions.

Composition-dependent structural transitions have previously been reported in MAG-based mixed lipid systems, although studies specifically addressing the partial replacement of MAGs with DAGs remain limited [10,15,34,35]. Borné et al. investigated the phase behavior of monoolein/diolein mixtures and demonstrated that partial replacement of monoolein with diolein markedly altered the phase behavior, destabilizing the bicontinuous cubic phase while expanding the stability region of the inverse hexagonal phase [40]. Similar composition-dependent structural transitions have also been observed in MAG-based nanodispersions containing fatty acids or other lipid components [10,15,21]. More recently, the importance of DAG molecular structure was demonstrated in pH-responsive nano-self-assemblies containing 2-hydroxyoleic acid and either 1,2-diolein, 1,3-diolein, or mixtures of the two positional isomers, where the DAG structure and positional isomerism influenced the resulting internal nanostructure [38].

These observations are consistent with fundamental biophysical studies showing that DAG incorporation can strongly influence lipid spontaneous curvature and promote inverse nonlamellar structures [29,41,42]. Leikin et al. demonstrated that incorporation of DAGs increases the magnitude of negative spontaneous curvature, while Szule et al. showed that the curvature-promoting effect of DAGs depends on their molecular structure, including acyl-chain length and degree of unsaturation [41,42]. Basanez et al. further reported that relatively small amounts of DAG can facilitate transitions toward inverse nonlamellar structures [29].

The present MLO/DLO system follows the same general principle that lipid composition governs interfacial packing and internal mesophase organization, while differing from previously reported systems in an important respect. Here, a fully polyunsaturated MAG/DAG pair composed of highly pure monolinolein and dilinolein was investigated while temperature, aqueous medium, total lipid concentration, stabilizer concentration, and preparation conditions were kept constant. This allowed the influence of the MLO:DLO ratio to be evaluated independently of other formulation variables. Under these conditions, increasing the DLO fraction progressively destabilized the ordered inverse hexagonal nanostructure and resulted in the appearance of an L_2_-type scattering profile, with the H_2_ phase retained only at the highest MLO content investigated.

At certain lipid compositions, the colloidal transformations from cubosomes via hexosomes toward more highly disordered inverse structures may also proceed through micellar cubosomes (nanoparticles with an internal inverse-micellar cubic *Fd3m* phase) [35]. This discontinuous (micellar) cubic phase is typically observed within narrow temperature- or composition-dependent regions in the phase diagrams of systems based on different lipids with nonlamellar phase-forming propensities [35,39,43]. In the present MLO/DLO nanodispersion, however, no intermediate cubic *Fd3m* phase was detected; instead, the SAXS profiles at the investigated lipid compositions indicated a composition-dependent transformation from hexosomes directly toward nanoparticles with highly disordered internal nanostructures. For example, Yaghmur et al. demonstrated that incorporation of tetradecane into MLO systems induced transformations from ordered cubic and hexagonal mesophases toward disordered inverse micellar structures as interfacial curvature increased [15], showing a structural trend similar to the composition-dependent H_2_-to-disordered transition observed in the present MLO/DLO system. These observations further suggest that the H_2_ phase remains stable over a relatively narrow composition range in the MLO/DLO system. While the 90:10 MLO/DLO formulation exhibited a well-defined inverse hexagonal nanostructure, increasing the DLO content by only 10 wt% resulted in the loss of long-range H_2_ order and the appearance of a broad SAXS correlation maximum. Experimental studies have further shown that long-chain unsaturated DAGs increase the magnitude of negative spontaneous curvature and thereby influence the balance between competing inverse mesophases [29,41,42]. Together, these findings suggest that progressive replacement of MLO by DLO shifts the preferred interfacial packing toward structures requiring increasingly negative curvature. Within the investigated composition range, the H_2_ phase was retained only at the highest MLO content, indicating that its compositional stability in the MLO/DLO system is relatively limited. However, since no intermediate compositions between pure MLO and the 90:10 MLO/DLO formulation were included in the experimental series, the existence of a very narrow cubic-phase region at DLO contents below 10 wt% cannot be excluded. Therefore, the present results indicate a direct loss of long-range H_2_ order and emergence of a highly disordered internal phase within the investigated composition range, rather than demonstrating that the MLO/DLO system intrinsically bypasses a cubic mesophase.

The structural characterization in the present study is based primarily on SAXS, complemented by cryo-TEM observations. SAXS provides direct information on the presence or absence of long-range periodic order, whereas cryo-TEM provides complementary morphological information on the nanoparticles and their internal nanostructures. Environment-sensitive fluorescent probes such as Nile Red can provide information on changes in local polarity and hydrophobic environments associated with structural transitions in lipid assemblies [44]; however, such measurements do not, on their own, provide an unambiguous assignment of an inverse-micellar phase. Moreover, DLO’s molecular geometry, a diacylglycerol bearing two linoleoyl chains and a minimal effective headgroup area (Figure 1A), is geometrically incompatible with the positive curvature required for a normal micelle (see Section 2 above), making a probe assay designed to distinguish normal from inverted micelles unlikely to be particularly discriminating for the present system. The remaining structural ambiguity here lies instead between two similarly nonpolar disordered inverse morphologies, a highly disordered L_2_-type nanostructure and nanoemulsion droplets, rather than between a normal and an inverted micelle. Other techniques, including small-angle neutron scattering (SANS) and pulsed-gradient spin-echo NMR (PGSE-NMR), may provide further structural information [44,45]. Such complementary approaches would be valuable in future studies aimed at further characterizing the highly disordered internal organization of DLO-rich MLO/DLO nanodispersions.

Overall, these findings demonstrate that DLO incorporation strongly modulates the confined internal self-assembled architecture of Pluronic F127-stabilized MLO-based nanodispersions, enabling a lipid composition-driven transformation from hexosomes with a well-defined internal H_2_ phase toward nanoparticles with highly disordered internal nanostructures. This lipid compositional sensitivity provides a structural basis for designing nonlamellar liquid crystalline nanocarriers with composition-controlled internal architectures, where designated colloidal transformations between different internal inverse organizations may alter the connectivity and dimensions of aqueous and hydrophobic nanodomains and thereby influence drug-loading capacity and release behavior.

Although the present study focused on the composition-dependent internal nanostructure of freshly prepared MLO/DLO nanodispersions, the potential influence of oxidative stability on the long-term structural integrity of these systems also deserves consideration. Both MLO and DLO contain unsaturated linoleoyl (C18:2) chains, and unsaturated lipids are inherently susceptible to oxidative degradation, which may lead to the formation of oxidation products, including lipid hydroperoxides [46]. Lipid oxidation during prolonged storage may therefore affect the structural features of these nanodispersions. Systematic investigation of the relationship between DLO content, oxidative stability, and long-term structural integrity represents an important direction for future studies.

### 2.2. Morphological Characteristics of MLO/DLO Nano-Self-Assemblies

**Figure 2:**
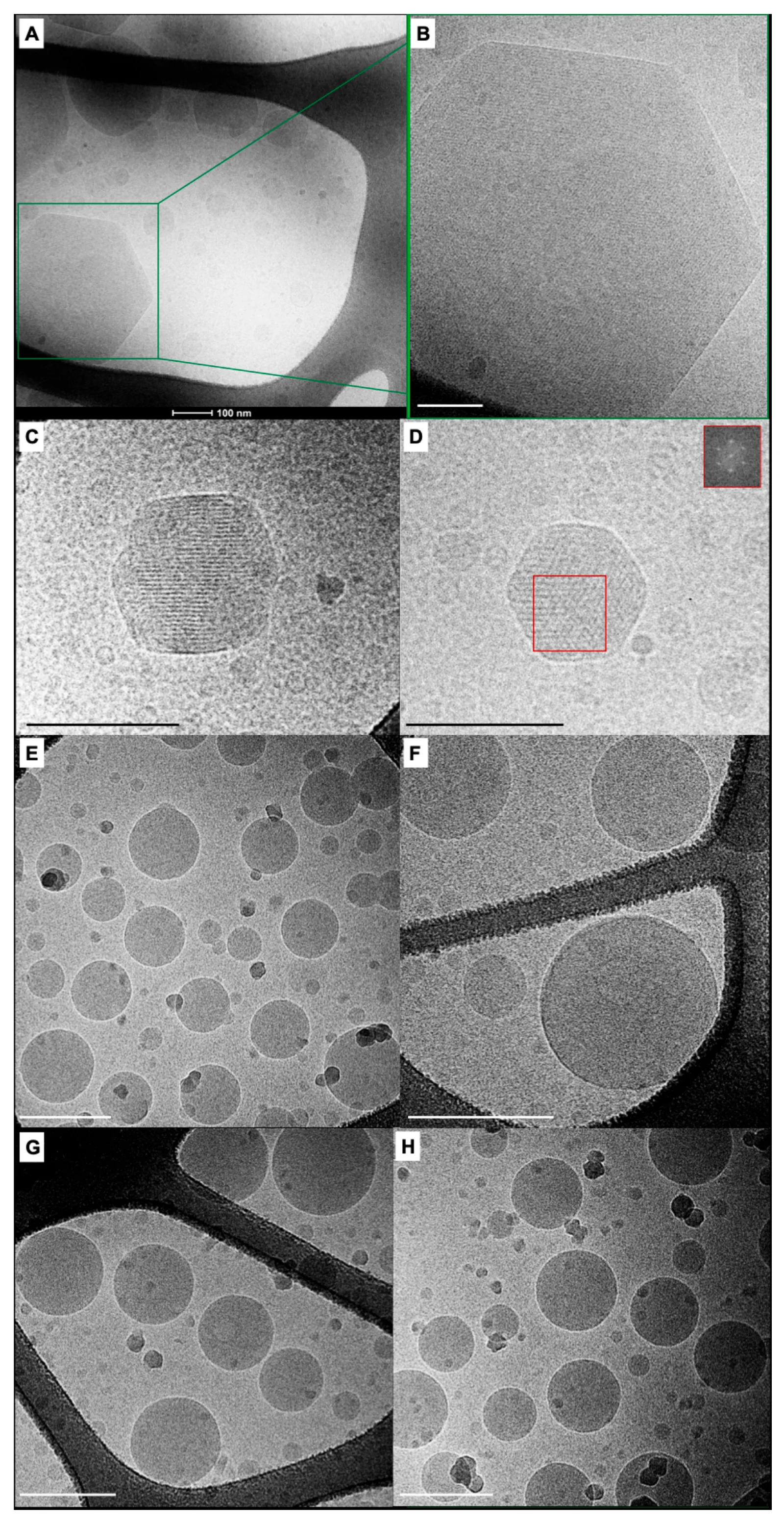
Cryo-TEM characterization of MLO/DLO nanodispersions. (**A**) Low-magnification overview image showing faceted, polygonal nanoparticles characteristic of hexosomes. The green boxed region is enlarged in (**B**), revealing the internal structural features of the particle. (**C**) High-magnification image showing well-defined lattice fringes appearing as curved striations, consistent with locally ordered cylindrical domains in the internal H_2_ phase. (**D**) Representative particle selected for fast Fourier transform (FFT) analysis. The red square indicates the region selected for FFT, and the corresponding inset shows discrete diffraction spots with hexagonal symmetry, from which a real-space periodicity of approximately 4.3 nm was estimated, in reasonable agreement with the first-order H_2_ periodicity determined by SAXS. (**E**,**F**) DLO-rich nanodispersion (sample MD_5_, MLO:DLO 50:50, *w*/*w*) and (**G**,**H**) the control nanodispersion prepared from DLO alone (sample MD_10_, MLO:DLO 0:100, *w*/*w*), showing predominantly spherical nanoparticles with a dense lipidic core lacking the periodic internal striations observed in (**C**). Dark bands in (**A**,**F**,**G**) correspond to bars of the lacey carbon support grid and the small, very dark angular features in (**H**) correspond to crystalline ice contamination rather than nanoparticle substructure. Scale bars: (**A**,**C**–**H**) 100 nm, (**B**) 10 nm.

Cryo-TEM was used as a direct imaging method to gain insight into the nanoparticle morphology and the internal self-assembled architecture of selected MLO/DLO nanodispersions, complementing the SAXS analysis. In agreement with the SAXS analysis, the micrographs of the MLO-rich nanodispersion (sample MD_1_), prepared at an MLO:DLO weight ratio of 90:10 (*w*/*w*), displayed faceted, polygonal nanoparticles with clearly resolved typical internal structural features of internal H_2_ phase (Figure 2). These colloidal objects exhibited alternating dark and bright striations arranged over locally ordered domains, consistent with projections of periodically organized cylindrical domains in the internal H_2_ phase and supporting their assignment as hexosomes [9].

To further support the local assignment of the internal H_2_ nanostructure, fast Fourier transform (FFT) analysis was performed on a selected high-resolution area within the individual nanoparticle (Figure 2). The corresponding FFT patterns showed discrete maxima arranged with approximate hexagonal symmetry, confirming the presence of locally ordered domains. The real-space periodicity estimated from the FFT analysis was approximately 4.3 nm, in reasonable agreement with the first-order hexagonal interplanar spacing (*d*_100_ ≈ 4.56 nm) derived from SAXS. The corresponding hexagonal lattice parameter, calculated from *d*_100_, was *a* = 5.27 nm. This agreement between real-space FFT analysis and reciprocal-space SAXS measurement supports the assignment of sample MD_1_ as hexosomes.

In addition to the dominant hexosome population, a minor coexisting fraction of vesicle-like spherical structures of varying size were observed in different cryo-TEM images (marked with white arrows, Supplementary Figure S1). Such coexisting colloidal nano-objects reflect the inherent morphological heterogeneity of nonlamellar lipid nanodispersions, where nanoparticle subpopulations with different morphologies and internal architectures may coexist depending on lipid type and composition, stabilizer type and concentration, and preparation conditions [9,16,47].

To gain further insight into the structural evolution associated with increasing DLO content, additional cryo-TEM observations were performed on two nanodispersions containing relatively high DLO contents (MD_5_ and MD_10_, Figure 2E,F and Figure 2G,H, respectively) and displaying highly disordered SAXS profiles. In contrast to the faceted hexosomes observed for MD_1_, the additional cryo-TEM micrographs revealed predominantly spherical nanoparticles, characterized by an intense lipid core and discernible internal nanoscale features. Unlike the well-defined periodic striations observed within the MD_1_ hexosomes, no clearly resolved long-range periodic internal organization was evident in these DLO-rich nanoparticles. The cryo-TEM observations therefore indicate a pronounced change in nanoparticle morphology and internal organization upon increasing DLO content. However, the internal nanostructures are not sufficiently resolved to allow full characterization and unambiguous assignment of the internal phase. Taken together with the SAXS findings, these observations indicate a colloidal transformation from hexosomes with a well-defined internal H_2_ phase toward nanoparticles with a highly disordered internal phase as the DLO content increases.

### 2.3. Particle Size Analysis of MLO/DLO

DLS was used to determine the hydrodynamic diameter (size) and size distribution, expressed as the polydispersity index (PDI), of MLO/DLO nanodispersions prepared at different lipid compositions (Table 1). The 90:10 MLO:DLO nanodispersion displayed the largest mean hydrodynamic diameter, 288.53 ± 21.39 nm, together with the highest PDI value, 0.31 ± 0.01. Progressive replacement of MLO by DLO led to smaller nanodispersions with lower PDI values, indicating that the size characteristics were strongly affected by lipid composition.

The decrease in mean hydrodynamic nanoparticle size was most pronounced upon increasing the DLO content from 10 to 50 wt%. The mean nanoparticle size decreased from 288.53 ± 21.39 nm for the 90:10 MLO:DLO nanodispersion to 201.73 ± 1.78 nm for the 80:20 MLO:DLO nanodispersion and reached values of approximately 157–164 nm for compositions between 50:50 and 30:70 MLO:DLO. These changes in size characteristics are most likely associated with the colloidal transformations described above, where progressive replacement of MLO by DLO induced colloidal transformation from hexosomes toward nanoparticles with highly disordered internal nanostructures. At higher DLO contents, the mean hydrodynamic nanoparticle size moderately increased, reaching 169.07 ± 2.46 nm for the 20:80 MLO:DLO nanodispersion, 180.90 ± 1.04 nm for the 10:90 MLO:DLO nanodispersion, and 199.25 ± 0.21 nm for the nanodispersion produced from DLO alone.

It is seen that at high DLO contents, a moderate increase in hydrodynamic diameter was observed despite the continued structural transition toward increasingly disordered internal nanostructures. Similar nonmonotonic particle size trends have previously been reported for mixed lipid nanodispersions. Nakano et al. observed that particle size initially decreased and subsequently increased with increasing oleic acid content and attributed this behavior to composition-dependent changes in the physicochemical properties of the lipid core [48]. Likewise, Tran et al. reported that particle size decreased during the transition from cubic nanoparticles to hexosomes but increased again upon formation of L_2_-type emulsified microemulsions [49]. Together, these observations suggest that the hydrodynamic diameter of mixed lipid nanodispersions is influenced not only by the internal mesophase but also by the physicochemical characteristics of the dispersed lipid phase. In the present MLO/DLO system, the moderate increase in particle size at high DLO contents may therefore reflect gradual changes in the physicochemical and interfacial characteristics of the increasingly DLO-rich dispersed phase, rather than the re-establishment of long-range internal ordering. The consistently low PDI values of these formulations further support that the observed size increase is unlikely to result from pronounced particle aggregation.

The PDI values were relatively low for most DLO-containing nanodispersions, ranging from 0.06 to 0.19, compared with 0.31 ± 0.01 for the 90:10 MLO:DLO nanodispersion. Overall, the DLS results show that progressive replacement of MLO by DLO altered the mean hydrodynamic nanoparticle sizes of MLO-based nanodispersions while maintaining nanoparticle sizes below 300 nm under the investigated experimental conditions.

## 3. Materials and Methods

### 3.1. Materials

Monolinolein (MLO, with a purity of >99%) and dilinolein (DLO, with a purity of >99%) were purchased from Larodan (Stockholm, Sweden). Pluronic F127 was a gift from BASF SE (Ludwigshafen, Germany). Phosphate-buffered saline (PBS, 10 mM) at pH 7.4 was prepared using Milli-Q water (Millipore Direct-Q3 UV system, Billerica, MA, USA), sodium chloride, potassium chloride, dibasic sodium phosphate, and monobasic potassium phosphate from Sigma-Aldrich (St. Louis, MO, USA). These ingredients were of analytical grade and used as received without further purification.

### 3.2. Preparation of MLO/DLO-Based Aqueous Nanodispersions

MLO/DLO-based aqueous nanodispersions were prepared at a constant total lipid content of 5.0 wt% (binary mixture of MLO and DLO) and 1.0 wt% Pluronic F127. A series of formulations was prepared at MLO:DLO weight ratios ranging from 0:100 to 100:0 in 10% increments. Briefly, the required amounts of MLO and DLO were mixed thoroughly in glass vials at 25 °C to obtain homogeneous binary lipid mixtures. Each mixture was then dispersed in phosphate-buffered saline (PBS, 10 mM, pH 7.4) containing 1.0 wt% Pluronic F127. The emulsification process was performed using an ultrasonic processor (Qsonica 500, Qsonica LLC, Newtown, CT, USA) operating at 30% of its maximum power for 5 min in pulse mode (5 s pulses with 2 s breaks) as previously described [21]. No pH adjustment was applied during or after emulsification. The freshly prepared dispersions were flushed with nitrogen gas for 2 min to minimize oxidation, sealed, covered with aluminum foil, and stored at 25 °C until further analysis. No macroscopic phase separation or aggregation was visually observed during storage over at least one month at 25 °C.

### 3.3. Synchrotron Small-Angle X-Ray Scattering (SAXS)

The structural characterization of MLO/DLO-based nanodispersions was carried out at the Austrian SAXS beamline of the ELETTRA synchrotron light source (Trieste, Italy). Measurements were performed at 25 °C using an X-ray beam with a wavelength of 1.54 Å and an energy of 8 keV. The sample-to-detector distance was set to 1314 mm, covering a *q*-range of 0.07–5.0 nm^−1^ (*q* = 4*π sin* (*θ*)/*λ*). Silver behenate (CH_3_–(CH_2_)_20_–COOAg; *d* = 58.38 Å) was used as a calibration standard. The two-dimensional SAXS patterns were collected using a Pilatus 1 M detector (Dectris Ltd., Baden, Switzerland) and radially integrated into one-dimensional scattering profiles *I(q)* using Fit2D software.

Lorentzian fitting was applied to determine the *q*-values of the detected Bragg reflections. The *d*-spacing values were calculated from the SAXS reflections using the equation *d* = 2*π*/*q*.

### 3.4. Cryo-Transmission Electron Microscopy (Cryo-TEM)

Cryo-TEM analysis was performed on the MLO-rich formulation (90:10 MLO:DLO, *w*/*w*), which was selected based on the SAXS results showing the presence of a well-defined inverse hexagonal (H_2_) phase. To further probe the internal nanostructure at higher DLO content, additional cryo-TEM imaging was performed under identical conditions on the DLO-rich nanodispersions MD_5_ (MLO:DLO 50:50, *w*/*w*) and MD_10_ (MLO:DLO 0:100, *w*/*w*). The morphology of the nanodispersions was examined under frozen-hydrated conditions as previously described [38,50]. Briefly, 3–4 μL of each nanodispersion was deposited onto a lacey carbon 300-mesh copper grid (Ted Pella Inc., Redding, CA, USA) that had been glow-discharged prior to use. The excess liquid was blotted using a Vitrobot IV system (FEI, Eindhoven, The Netherlands) with a blotting time of 5 s, blotting force 0, and 100% humidity at 4 °C. The grids were rapidly plunged into liquid ethane cooled by liquid nitrogen (−180 °C) to achieve vitrification.

Cryo-TEM imaging was performed using a Tecnai G2 20 transmission electron microscope (FEI, Eindhoven, The Netherlands) operated at 200 kV under low-dose conditions (~5 e^−^/Å^2^). A Gatan 626 cryo-holder (Gatan, Inc., Pleasanton, CA, USA) was used to maintain the vitrified state during imaging. The digital micrographs were analyzed using ImageJ software (version 1.54g; National Institutes of Health, Bethesda, MD, USA) and fast Fourier transform (FFT) analyses were performed on selected regions to evaluate internal nanostructural ordering within the particles.

### 3.5. Dynamic Light Scattering (DLS)

The hydrodynamic diameter and polydispersity index (PDI) of the MLO/DLO-based nanodispersions were determined by dynamic light scattering (DLS) using a Zetasizer Nano ZS90 (Malvern Panalytical Ltd., Malvern, UK) equipped with a 4 mW He-Ne laser operating at 633 nm and a fixed scattering angle of 90°. Measurements were performed at 25 °C after diluting the samples 1:10 (*v*/*v*) with phosphate-buffered saline (PBS, 10 mM, pH 7.4). Each sample was measured in triplicate, and the results were expressed as mean ± standard deviation (SD).

## 4. Conclusions

MLO/DLO-based aqueous nanodispersions provide a simple compositionally tunable platform for modulating the internal self-assembled architecture of lipid nanoparticles. Systematic variation in the lipid composition induced a direct colloidal transformation from hexosomes with a well-defined internal H_2_ phase toward nanoparticles with a highly disordered internal phase. SAXS showed loss of the characteristic H_2_ Bragg reflections upon increasing DLO content and the appearance of broad correlation maxima associated with short-range internal structural correlations. Complementary cryo-TEM observations further revealed a transition from faceted hexosomes with clearly resolved internal H_2_ nanostructures to predominantly spherical nanoparticles characterized by an intense lipid core and less clearly resolved internal nanoscale features.

Taken together, SAXS and cryo-TEM observations indicate a composition-driven colloidal transformation from nanoparticles with a well-defined internal H_2_ phase toward nanoparticles with a highly disordered internal phase as the DLO content increases. However, the internal nanostructures of the DLO-rich nanoparticles could not be sufficiently resolved by cryo-TEM to allow full characterization and unambiguous assignment of the internal phase. The observed structural evolution is compatible with increasingly negative spontaneous curvature induced by DLO incorporation and may involve an L_2_-like inverse-micellar organization, among other possible disordered inverse arrangements such as nanoemulsion droplets. Overall, these findings show that relatively small changes in MAG/DAG composition can strongly influence the internal nanostructure and colloidal characteristics of lipid nanodispersions. This composition-dependent control provides a practical route for tailoring internally self-assembled lipid nanocarriers through lipid molecular design rather than external triggers such as temperature variation.

From a nanocarrier design perspective, these findings highlight lipid composition as a useful design variable for directing colloidal transformations in self-assembled lipid nanodispersions. The MLO/DLO system therefore offers a model platform for studying how changes in lipid composition influence nanoparticle self-assembled architecture. Such composition-guided tuning may support the design of lipid nanocarriers with tunable internal structural features for modulating drug loading and release behavior.

## Figures and Tables

**Table 1:** Composition, structural parameters, and colloidal characteristics of MLO/DLO nanodispersions. The hydrodynamic diameter and polydispersity index (PDI) were obtained from dynamic light scattering (DLS) measurements. The *d*-spacing values were calculated from SAXS data using *d* = 2*π*/*q*. The hyphen (“-”) indicates that no *d*-spacing value is reported. For MD_1_, this reflects the use of hexagonal indexing to obtain the lattice parameter (*a* = 5.27 nm) rather than a single correlation maximum. For the remaining samples, no distinct or sufficiently intense correlation feature could be identified from which a reliable *d*-spacing could be extracted (see Section 2.1 for details). Values are expressed as mean ± SD (*n* = 3).

Name	MLO:DLO (wt%)	*d*-Spacing (nm)	Hydrodynamic Diameter (nm)	PDI
MD_1_	90:10	-	288.53 ± 21.39	0.31 ± 0.01
MD_2_	80:20	4.527	201.73 ± 1.78	0.19 ± 0.01
MD_3_	70:30	4.075	165.40 ± 0.27	0.12 ± 0.02
MD_4_	60:40	3.617	162.10 ± 0.55	0.08 ± 0.01
MD_5_	50:50	3.454	157.07 ± 1.25	0.07 ± 0.02
MD_6_	40:60	3.185	159.60 ± 0.63	0.07 ± 0.01
MD_7_	30:70	2.873	163.63 ± 0.72	0.06 ± 0.02
MD_8_	20:80	-	169.07 ± 2.46	0.09 ± 0.02
MD_9_	10:90	-	180.90 ± 1.04	0.09 ± 0.01
MD_10_	0:100	-	199.25 ± 0.21	0.10 ± 0.01

## Data Availability

The data presented in this study are available from the corresponding author upon reasonable request.

## Supplementary Materials

The following supporting information can be downloaded at: https://www.mdpi.com/article/10.3390/molecules31173031/s1, Figure S1: Additional cryo-TEM micrographs of the 90:10 MLO:DLO (MD_1_) nanodispersion showing vesicle-like spherical structures coexisting with the dominant hexosome population.
